# Platelets in COVID-19 disease: friend, foe, or both?

**DOI:** 10.1007/s43440-022-00438-0

**Published:** 2022-12-03

**Authors:** Marta Smęda, Ebrahim Hosseinzadeh Maleki, Agnieszka Pełesz, Stefan Chłopicki

**Affiliations:** 1 Jagiellonian Centre for Experimental Therapeutics (JCET), Jagiellonian University, Krakow, Poland; 2 Department of Pharmacology, Jagiellonian University Medical College, Krakow, Poland

**Keywords:** COVID-19, Platelets, Endothelial barrier, Pulmonary circulation

## Abstract

Immuno-thrombosis of COVID-19 results in the activation of platelets and coagulopathy. Antiplatelet therapy has been widely used in COVID-19 patients to prevent thrombotic events. However, recent analysis of clinical trials does not support the major effects of antiplatelet therapy on mortality in hospitalized COVID-19 patients, despite the indisputable evidence for an increased risk of thrombotic complications in COVID-19 disease. This apparent paradox calls for an explanation. Platelets have an important role in sensing and orchestrating host response to infection, and several platelet functions related to host defense response not directly related to their well-known hemostatic function are emerging. In this paper, we aim to review the evidence supporting the notion that platelets have protective properties in maintaining endothelial barrier integrity in the course of an inflammatory response, and this role seems to be of particular importance in the lung. It might, thus, well be that the inhibition of platelet function, if affecting the protective aspect of platelet activity, might diminish clinical benefits resulting from the inhibition of the pro-thrombotic phenotype of platelets in immuno-thrombosis of COVID-19. A better understanding of the platelet-dependent mechanisms involved in the preservation of the endothelial barrier is necessary to design the antiplatelet therapeutic strategies that inhibit the pro-thrombotic activity of platelets without effects on the vaso-protective function of platelets safeguarding the pulmonary endothelial barrier during multicellular host defense in pulmonary circulation.

## Introduction

The characteristic feature of severe acute respiratory syndrome coronavirus 2 (SARS-CoV-2) infection (COVID-19 disease) is severe lung dysfunction which may progress to acute respiratory distress syndrome (ARDS) [1]. One of the main predisposing factors for the severe course of COVID-19 is increasing patient age, which is associated with higher expression of angiotensin-converting enzyme 2 (ACE-2) (a receptor for the SARS-CoV-2 spike protein), immune dysregulation, changes in microbiota, lower levels of steroid hormones, and senescence-associated oxidative stress [2]. Aging is also a major risk factor for the development of cardiovascular diseases, which predisposes to a severe course of COVID-19 [3]. Both healthy aging as well as age-related diseases are characterized by endothelial dysfunction, which increases the probability of a severe COVID-19 course [4–6]. Moreover, in the context of SARS-CoV-2 infection, pre-existing age- and/or disease-associated endothelial dysfunction could be rapidly exacerbated [7]. Therefore, it is not surprising that a biomarker of endothelial activation and altered endothelial permeability Angiopoietin-2 was found to be a good predicting factor for severe cases of COVID-19 disease [8]. In fact, COVID-19 was suggested to represent *an endothelial disease* [9]. Accordingly, endothelial phenotype is an important determinant of the outcome of COVID-19-related organ injury.

In athero-thrombosis, the pathophysiology of endothelial dysfunction is complex [10, 11]. Importantly, it is invariably linked to low-grade inflammation, the impairment of antiplatelet endothelial mechanisms [12], and the hyper-activation of platelets. Accordingly, antiplatelet therapy represents a major pharmacological strategy aimed to reduce the risk of athero-thrombosis-associated cardiovascular events, and there is little doubt about the clinical efficacy of antiplatelet drugs in these indications.

Similarly, hyper-activated platelets, together with pronounced and dysregulated inflammation and coagulopathy, represent hallmarks of COVID-19; however, antiplatelet therapy, surprisingly, has not provided clear clinical benefits in COVID-19 patients [13]. Indeed, despite some positive results from early observational studies, in a number of recent randomized clinical trials (RCTs), antiplatelet therapy did not affect the progression of COVID-19 (ACTIV-4B, RECOVERY, ACTIV-4A, REMAP-CAP) [14]. Accordingly, despite strong evidence for an increased risk of thrombotic complications in COVID-19 disease, these clinical trials do not suggest any significant effects of antiplatelet therapy on mortality in hospitalized COVID-19 patients. This apparent paradox requires explanation and might suggest that the non-hemostatic function of platelets is important in the course of COVID-19. Indeed, platelets have an important role in sensing and orchestrating host response to infectious agents. Platelets can act as pathogen sensors within the blood [15], and a number of platelet-dependent mechanisms related to host defense response have recently been documented that are not directly related to the hemostatic function of platelets [16–18]. These non-hemostatic immunological functions of platelets and platelet-mediated immunity might be of key importance in the host response to infection and even in host survival during viral infection [19]. Furthermore, during host defense occurring in circulation, platelets have an additional important role to play: they safeguard endothelial barrier integrity. Accordingly, antiplatelet therapy might result in the disruption of the platelet-dependent regulation of endothelial barrier integrity and the inhibition of mechanisms of inflammation-associated hemostasis [20]. These mechanisms are of particular importance in the lungs [21–24] which also represent a major organ affected by severe COVID-19 disease.

In this review, we propose that one possible explanation for the possible lack of efficacy of antiplatelet drugs in COVID-19 is related to the importance of fully functional platelets in the fight against invading viruses during host defense in the circulation. In this context, we briefly summarize the hemostatic and non-hemostatic function of platelets. In particular, we discuss recently discovered platelet-dependent mechanisms that protect the endothelial barrier during inflammation in the processes that have been termed as non-classical inflammation-associated hemostasis [20]. It might well be that the inhibition of platelet function, if affecting the protective aspect of platelet activity, might diminish clinical benefits resulting from the inhibition of the pro-thrombotic phenotype of platelets in immuno-thrombosis of COVID-19.

## Platelets in thrombosis and hemostasis; antiplatelet drugs in cardiovascular diseases

The most recognized and well-described function of platelets is related to classical hemostasis [25] involving mechanisms designed to prevent blood loss after vascular injury; this may, however, lead to pathological thrombosis in arterial or in the venous circulation and to myocardial infarction, or venous and pulmonary thromboembolism, respectively [26, 27]. Although platelets are key players in arterial thrombosis, there is also increasing evidence that platelets have a role in forming venous thrombi [28–31].

**Fig. 1:**
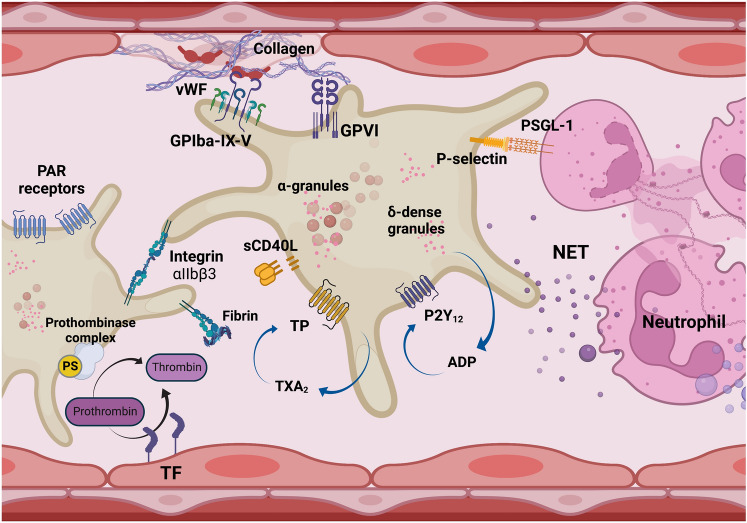
Hemostatic function of platelets. Following endothelial cell injury, the von Willebrand factor (vWF) via the platelet-receptors GPIbαIX-V complex initiates platelet adhesion, which also involves interactions of sub-endothelial collagen and GPVI receptors on platelets, platelet–platelet aggregation via the αIIbβ3 integrin receptor, and thrombin activation leading to thrombus formation. Thrombin is also a strong platelet activator by binding to the protease-activated receptor (PAR) [41, 42]. COX-1-derived thromboxane A2 production and ADP release amplify platelet activation via TP and P2Y_12_ receptors, respectively. The thrombus grows by the deposition of more fibrin, and the accumulation of red blood cells and leukocytes including neutrophils. Activated platelets release pEVs, which induce neutrophil activation and subsequent NET formation. Platelet–neutrophil interactions playing a key role in the host response are also involved in the pathogenesis of thrombosis. Platelets represent a major source of sCD40L, orchestrate inflammatory response and are also involved in the activation of endothelial inflammation [43]. The pro-thrombotic function of platelets is closely interlinked with host defense and inflammatory responses

Platelets rapidly respond to vascular injury and, by platelet tethering and firm attachment with activated endothelium—platelet degranulation, aggregation, and platelet plug is formed [32, 33] with subsequent activation of thrombin and thrombus formation. The mechanisms involve binding of the GPIb–IX–V complex on platelets to immobilized von Willebrand factor, platelet GPVI binding to collagen, amplification of platelet activation by COX-1-derived thromboxane A_2_ production and ADP release from platelet granules, the activation of the platelet αIIbβ3 receptor, prothrombinase complex formation and thrombin generation resulting in fibrin formation (Fig. 1). The hemostatic function of platelets involves interlinked processes of platelet activation with the activation of the coagulation pathway [34, 35]. The excessive activation of these mechanisms leads to pathological thrombosis which is the cause of cardiovascular pathology [36]. Hence, antiplatelet and anticoagulant treatment represents the mainstay of treatment of cardiovascular diseases, such as unstable coronary artery disease or myocardial infarction, with the efficacy of such a treatment strategy established in decreasing cardiovascular mortality [37]. In particular, the beneficial role of antiplatelet therapy in coronary artery disease has been well-established with ADP antagonists (clopidogrel, ticagrelor, and prasugrel inhibiting the P2Y_12_ receptor) and aspirin (acetylsalicylic acid)-inhibiting TXA_2_ synthesis through cyclo-oxygenase-1 (COX-1) blockade, which are used as dual or single therapy depending on clinical conditions. Given the clinical efficacy of antiplatelet agents, there are various novel antiplatelet strategies being developed or studied, e.g., vorapaxar PAR1 receptor antagonists, GP (glycoprotein) Ib-IX-V or GPVI antagonists, protein disulfide isomerase-PDI inhibitors, carbon monoxide releasing molecules (CORMs), or others [12]. A combination of various antiplatelet drugs and anticoagulants is beneficial and there is little doubt about the clinical benefit of such a strategy to prevent cardiovascular events in patients with the acute coronary syndrome. The Holy Grail is to find novel combinations of antiplatelet and anticoagulant drugs that will afford protection and cause minimal bleeding, for example, using novel anticoagulants [38].

It is worth noting that the relationship between platelet activation and inflammation is well established in atherosclerosis and the efficacy of antiplatelet agents in cardiovascular diseases is not only ascribed to direct platelet inhibition and the inhibition of pro-thrombotic platelet activity, but also to the inhibition of the number of pro-inflammatory mediators released by activated platelets [39, 40].

## Platelets in COVID-19; antiplatelet drugs in COVID-19

In early reports on COVID-19, it was noted that this disease was associated with thrombocytopenia. In a number of later reports, platelet count was found to be a predictor of mortality, with low platelet counts significantly correlating with higher patient death rates [14], confirming the participation of platelets in COVID-19 pathogenesis and extensive platelet consumption in this disease [44]. Despite the fact that there is still some controversy regarding whether platelets express [45, 46] or do not express angiotensin-converting enzyme 2 (ACE-2) [47], a receptor for spike protein of SARS-Cov-2, thromboembolism was a common phenomenon in hospitalized COVID-19 patients [13], occurring with even higher prevalence than sepsis [48].

Indeed, patients with COVID-19 displayed a well-documented phenotype of hyper-activated platelets and autopsies report widespread micro-thrombi associated with neutrophil extracellular traps (NET), and with endothelial layer disruption in inflamed lungs [49–52]. A number of biomarkers reflecting platelet activation were found to be elevated in COVID-19 patients, and some of them displayed prognostic value. For example, increased plasma level of vWF was associated with disease severity and mortality in COVID-19 [53, 54]. Similarly, soluble P-selectin and PF4 were higher among severe COVID-19 patients [55, 56].

There is also a number of studies that characterized functionally hyper-activated phenotype of platelets from COVID-19 patients. Platelets from severely ill COVID-19 patients induced monocyte tissue factor expression (in a P-selectin and αIIb/β3 dependent manner) [57], which could amplify inflammation and coagulation in COVID-19. Increased platelet activation in the course of COVID-19 also led to the upregulated release of pro-inflammatory cytokines from platelets compared to platelets from healthy subjects [46]. Platelets from active COVID-19 displayed increased adhesion and aggregatory potential, and released more IL-1β and sCD40L in response to low levels of thrombin [46]. In a recent study, it was reported that platelets from severely ill and hospitalized COVID-19 patients exacerbated endotheliopathy via the release of the MRP8/14 protein, which activated endothelial cells, promoted their inflammatory hypercoagulable phenotype, that significantly correlated with poor clinical outcomes of COVID-19 patients [58].

Of note is that platelets represent a rich source of TGFβ, with nearly 50% of basal plasma concentration of TGFβ estimated to be derived from platelets [59]. It was reported that elevated TGFβ in COVID-19 patients [46] could contribute to lung damage. Since SARS-CoV-2 infection forms a favorable microenvironment for TGFβ activation upon its release from hyperactive platelets [60], platelet-derived TGFβ could significantly contribute to the pathogenesis of COVID-19 and lung failure. Therefore, TGFβ blockade was proposed as the potential treatment strategy for COVID-19 patients [61].

The hypercoagulable phenotype of platelets in COVID-19 patients can be transferred by platelet-derived extracellular vesicles (pEVs), which recapitulate most platelet functions associated with hemostasis and thrombus formation [62]. Importantly, pEVs can enter the tissues, transferring their content outside vasculature in the surroundings inaccessible to platelets [63] and can convey biological molecules, such as proteins, nucleic acids, and lipids, as well as surface markers, depending on the stimulus [64–66]. Indeed, it was shown that pEVs participate in viral spreading in COVID-19 [67] and numerous reports demonstrated that pEVs counts were higher in SARS-CoV-2-positive individuals [46, 68]. Since pEVs provide a pro-coagulant surface and the phosphatidylserine (PS) negatively charged surface of pEVs facilitates the formation of coagulation cascade complexes, their increased numbers can account, at least partially, for increased hypercoagulable events common in severe cases of COVID-19 disease. Paradoxically, phosphatidylserine (PS)-positive pEVs were increased only in non-severe cases of COVID-19, suggesting that pEVs could have been consumed in severe cases of COVID-19 due to excessive activation of coagulation [67, 69, 70]. The pEVs can also support COVID-19 thrombosis by platelet factor 4 (PF4) and high-mobility group box 1 protein (HMGB1) acting as damage-associated molecular patterns (DAMPs) [60, 71]. Yet another pEVs factor that could support COVID-19 thrombosis is tissue factor (TF), though its expression in both platelets and pEVs is still under debate [72]. Finally, viral-induced pEVs also enhanced NET formation and inflammatory reactions through TLR2 and CLEC5A-dependent mechanisms [73].

Altogether, there is overwhelming evidence that COVID-19 is related to increased platelet activation [74] and robust pEVs formation, pointing to the important role of platelet over-activation in the pathophysiology of COVID-19. The mechanisms that contribute to hyper-activated platelet phenotype and thrombi formation in response to infection with the SARS-CoV-2 virus involve direct interactions of the virus with platelets [75, 76], and a number of indirect mechanisms of platelet activation are linked to COVID-19 pathogenesis, such as endothelial cell damage [77–79], cytokine storm with its pro-coagulant consequences and others [80–82].

Given the emerging evidence of platelet hyper-activation and high prevalence of arterial and venous thromboembolism in hospitalized COVID-19 patients, anticoagulation treatment and antiplatelet agents have been widely used to reduce the risk of thrombotic events. Unexpectedly, clear evidence for the clinical benefit of antiplatelet treatment in COVID-19 is lacking [13, 83]. In the ACTIVE-4A clinical trial, the use of a P2Y_12_  antagonist in addition to a therapeutic dose of heparin, compared with a therapeutic dose of heparin only, did not improve outcomes of non-critically ill hospitalized COVID-19 patients [84]. Similarly, the ACTIVE-4B clinical trial did not confirm the benefits of aspirin or apixaban compared with placebo treatments for hospitalized COVID-19 patients [85]. The RECOVERY trial [65] was the largest randomized study investigating the effect of antiplatelet therapy for COVID-19, including 14,892 participants from 171 centers. An aspirin dose of 150 mg was added to standard care but did not reduce 28-day mortality or the probability of a severe course that would require invasive mechanical ventilation or lead to death. Moreover, based on the REMAP-CAP international adaptive platform trial, designed to determine the best treatment strategies for patients with severe pneumonia, antiplatelet therapy (aspirin, and P2Y_12_  anatgonists, such as clopidogrel, ticagrelor or prasugrel) alongside a prophylactic dose of anticoagulation in severe COVID-19 patients also did not show clinical benefits but increased the risk of major bleeding [86].

All these studies led to the conclusion that the beneficial effects of antiplatelet therapy in the progression of COVID-19 were uncertain [83]. Therefore, as recently summarized by Zong et al., even though there was an association between antiplatelet drug use and improved outcomes for COVID-19 patients reported in early observational studies, recently completed RCTs failed to confirm the effectiveness of antiplatelet treatment in preventing COVID-19 progression [14] (Table 1).

**Table 1:** Major randomized controlled trials (RCTs) investigating effects of antiplatelet treatment in COVID-19

No	Study	Drug	Results
1	Accelerating COVID-19 Therapeutic Interventions and Vaccines 4 Acute (ACTIV-4A trial) (*n* = 562)	87% of patients received a therapeutic dose of heparin by the end of study day 1. In the P2Y_12_ inhibitor group, ticagrelor was used in 63% of patients and clopidogrel in 37%	Among non-critically ill patients hospitalized for COVID-19, the use of a P2Y_12_ antagonist in addition to a therapeutic dose of heparin, compared with a therapeutic dose of heparin only, did not result in increased odds of improvement in organ support-free days within 21 days during hospitalization [84]
2	COVID-19 outpatient thrombosis prevention trial A Multi-center adaptive randomized placebo-controlled platform trial evaluating the efficacy and safety of anti-thrombotic strategies in COVID adults not requiring hospitalization at time of diagnosis (ACTIV-4B trial), (*n* = 657)	Apixaban 2.5 mg, Apixaban 5 mg, Aspirin (low-dose 81 mg), Placebo	Among symptomatic clinically stable outpatients with COVID-19, treatment with aspirin or apixaban compared with a placebo did not reduce the rate of a composite clinical outcome [85]
3	RECOVERY trial (*n* = 14,892)	Adult COVID-19 patients were randomly allocated in a 1:1 ratio to either the usual standard of care plus 150 mg aspirin once per day until discharge or the usual standard of care alone. The primary outcome was 28-day mortality	A similar number of COVID-19 patients with aspirin in addition to the usual standard of care group and the usual standard care group died within 28 days [87]
4	REMAP-CAP trial (*n* = 1549)	Treatment with an antiplatelet agent: aspirin or a P2Y_12_ inhibitor (clopidogrel, prasugrel, or ticagrelor) compared with no antiplatelet agent	Among critically ill patients with COVID-19, treatment with an antiplatelet agent, compared with no antiplatelet agent, had a low likelihood of providing improvement in the number of organ support—free days within 21 days [86]
5	Observational studies	Pooled 23 observational studies in a meta-analysis [88]	Antiplatelet treatment favored a lower risk of mortality [odds ratio (OR) 0.72, 95% confidence interval (CI) 0.61–0.85; *I*^2^ = 87.0%, *P* < 0.01]

Evidence from large randomized clinical trials listed as 1–4 did not confirm the therapeutic efficacy of antiplatelet drugs in severe COVID-19, while the rationale for using antiplatelet treatment in COVID-19 was initially supported by the result of the early observational studies (based on [88])

In accordance with these results, new recommendations by The International Society on Thrombosis and Hemostasis (ISTH) have been recently released [89] which highlight the limited benefit of antiplatelet therapy in COVID-19 patients. These recommendations draw attention to the fact that in hospitalized, non-critically ill COVID-19 patients, the addition of antiplatelet therapy may be even harmful. Of course, it could well be that antiplatelet therapy would be beneficial in some cases of COVID-19, for example, in patients with concomitant cardiovascular diseases or cardiovascular risk factors as indicated by a recent HOPE-COVID-19 trial [90]. Yet, in these circumstances, antiplatelet drugs most likely prevented the progression of cardiovascular disease triggered by COVID-19, rather than COVID-19 disease progression per se.

Altogether, the recent analysis of clinical trials does not support the major effects of antiplatelet therapy on mortality in hospitalized COVID-19 patients despite the indisputable evidence for an increased risk of thrombotic complications in COVID-19 disease. This apparent paradox requires explanation and might suggest that the non-hemostatic function of platelets, i.e., platelet-dependent mechanisms related to the host defense that are not directly related to the hemostatic function of platelets, might be of vital importance in the host response to infection [15–18].

## Versatile nature of platelets beyond classical hemostasis: immune response, resolution of inflammation and endothelial integrity

### Platelets and regulation of immune response

It is becoming increasingly clear that platelets are more versatile than initially thought and possess a significant variety of non-hemostatic immunological functions. Platelets are equipped to interact closely with bacteria and viruses and modulate the functions of a variety of cells of the innate and adaptive immune systems. Surprisingly, these ubiquitous blood components act as pathogen sensors in the blood, promoting pathogen elimination by a variety of mechanisms, and should be regarded as critical cells of the immune system [18, 91].

The most convincing evidence for the importance of platelet-mediated immunity comes from studies showing impaired pathogen removal and increased mortality in bacterial infections when platelet function was compromised [92, 93]. Similarly, it was shown that animals depleted of platelets had reduced viral clearance and an impaired virus-specific cytotoxic T lymphocyte (CTL)-dependent response [22]. The immune function of platelets is mediated by a variety of receptors that do not have an obvious function in hemostasis. Indeed, platelets have a plethora of membrane receptors able to detect pathogen- and danger-associated molecular patterns [PAMPs and DAMPs], such as Toll-like receptors [TLRs] and the C-type lectin receptors [CLRs]. It was shown that human platelet TLRs could recognize and discern various isoforms of bacterial lipopolysaccharide [LPS] via TLRs. Moreover, the expression and functionality of endosomal TLR-3, -7, and -9, able to respond to the viral genome, were detected in platelets [19, 94, 95]. For instance, platelet TLR7 could sense ssRNA and enhance the uptake of viruses (i.e., influenza virus) which lead to neutrophil NETosis [19, 96]. Platelets also express CLRs, recognizing bacterial, fungal, or viral glycans [97–99]. In particular, CLEC-2 is a CLR expressed on platelets [100, 101] and was identified to promote platelet activation [102]. A number of other receptors could be involved in platelet–pathogen interaction, including platelet integrins and cytokine receptors [103]. For instance, the b3 integrins are implicated in the binding of pathogenic hantaviruses [104] and C-X-C chemokine receptor (CXCR) type 4, and required co-receptors C–C chemokine receptor (CCR) type 1, 3 and 4 interact with human immunodeficiency virus (HIV) [105, 106].

Regarding SARS-CoV-2, as the mechanisms of interaction the virus with platelets are still controversial [46, 47], alternative receptors that could bind SARS-CoV-2 have been proposed, such as CD147, CD26, integrins and endosomal toll-like receptors-mediated pathway and others [107]. Platelets could be also activated through the FcγRIIA engagement of immune complexes (ICs) formed in patients with cross-reacting antibodies to SARS-CoV-2 [108] (shown to be involved in the H1N1 influenza virus interaction with platelets) [112].

Platelet-mediated immunity also involves antimicrobial products (collectively known as platelet microbicidal proteins (PMPs) [109]. Various families of PMPs are released from platelets including β-defensins (human β defensin 2), and kinocidins (CXCL4, CXCL7, and CCL5) [110]. The β-defensins are cationic antimicrobial peptides found in platelets, and they directly inhibit bacterial growth via membrane disruption and promote NETosis [111]. Platelet factor 4 (PF4) (also called the chemokine CXCL4) is an essential agent in leukocyte chemotaxis, but also exerts a direct antiviral activity. Surprisingly, activated platelets were able to eliminate the intra-erythrocytic malarial parasite *Plasmodium falciparum* 14 through a mechanism involving PF4 (CXCL4) and the erythrocyte Duffy antigen receptor (Fy) [112]. Platelets also secrete antimicrobial proteins such as PD1–PD4 (programmed cell death proteins 1–4) exhibiting antiviral properties against the cowpox virus [113]. Thrombocidins were initially purified from platelet granules and were characterized as truncated variants of the C-X-C chemokine, neutrophil-activating peptide-2 (NAP-2) [81]. They effectively eliminate several bacterial strains [114]. Interestingly, Campbell et al. found that interferon-induced transmembrane protein 3 (IFITM3), an antiviral immune gene, was significantly elevated in platelets from infected patients, and overexpression of IFITM3 in cultured MKs enhanced the MK’s resistance to DENV infection [115]. This work and the discovery of antiviral action of IFITM3 supplementation add specific antiviral platelet mechanisms to the rich repertoire of anti-antibacterial proteins in platelets with specific anti-antiviral mechanisms.

Although platelets have an arsenal of antibacterial and antiviral proteins, their major role in host defense seems to be linked to the sensing pathogens and, then, orchestrating pathogen removal by communicating with proficient cells of the innate response. Platelets interacting with neutrophils augment neutrophil function, bacterial trapping and bacterial removal [116, 117], increase ROS production and phagocytosis, as well as facilitate neutrophils extravasations [118]. Platelets interact with leukocytes via P-selectin, GPIbα, or αIIbβ3 [119].

Platelets influence the function of a variety of other cells of innate immune response: not only neutrophils but also monocytes, macrophages, eosinophils, and dendritic cells [120–123]. Mechanisms involve not only direct interactions forming heterotypic aggregates but also released factors. For example, platelet-derived cytokine IL-1β release in response to bacterial LPS or viral infection [124–126] leads to increased phagocytosis of bacteria and further IL-1β production by macrophages [91].

The pEVs can also regulate immune cell migration and immune defense mechanism by carrying a large variety of substances, such as various cytokines or chemokines (e.g., IL-1, RANTES), lipid mediators, enzymes, surface receptors such as CD40L, autoantigens, transcription factors, and competent respiratory mitochondria [16]. For instance, co-culture studies showed that platelets can induce the maturation of monocytes into macrophages [127], and platelet chemokine CXCL4 plays a significant role in this differentiation process [128]. Platelet cytokines, such as RANTES and IL-1β, have been shown to activate monocytes [129, 130]. Platelets have been implicated in the maturation and activation of dendritic cells [DCs], the most potent antigen-presenting cells, indicating that platelets can bridge the innate and adaptive immune systems. Activated platelets induce DC maturation in a CD40L-dependent manner [91]. Furthermore, platelets contribute to adaptive immunity via mechanisms depending on membrane receptors P-selectin, which support lymphocyte delivery to the peripheral lymph nodes [131], and by a CD40L-dependent mechanism which supports isotype switching by B cells, augments the CD8 + T cell response during viral infection and, consequently, supports specific immunoglobulin G (IgG) production [132, 133].

**Fig. 2:**
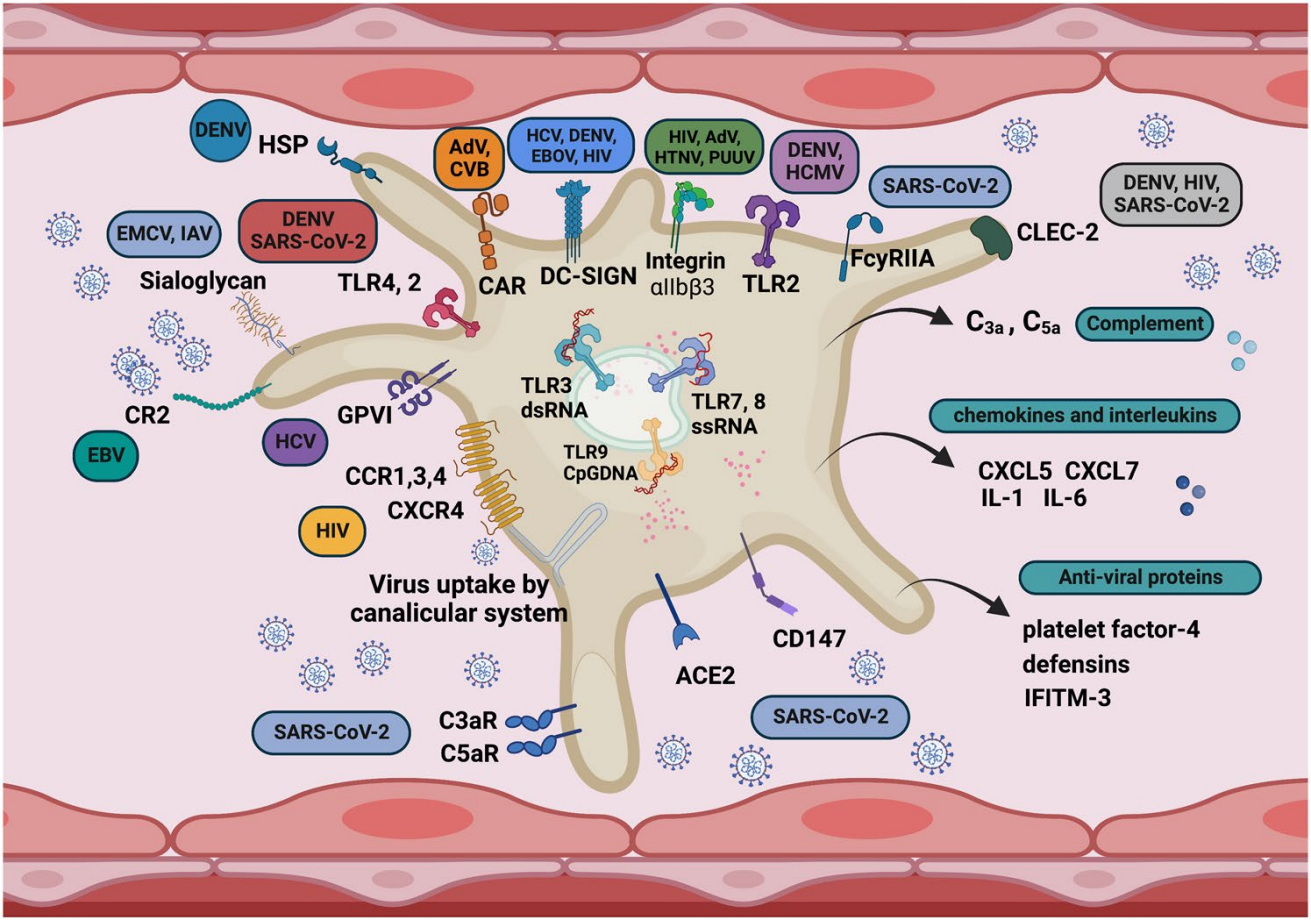
Non-hemostatic function of platelets; platelets as immune sentinels [15, 16]. Platelets are involved in pathogen recognition and communicating with a variety of immunological effector cells orchestrating host defense response [6, 15, 16, 18, 107, 134–139]. In particular, platelets express a number of receptors involved in virus binding and internalization, as documented for a number of viruses. Platelets express multiple pattern-recognition receptors and other receptors involved in innate response, including toll-like receptor (TLR) [95], C-type lectin domain family 2 and 5 (CLEC-2 and CLEC-5) [140], complement receptor (CR 3a and CR 5a), Cys-X-Cys (C-X-C) motif chemokine receptor type (CXCR1, CXCR1-2, and CXCR1-4) [105]. Platelets also express Fc gamma receptor II (FcγRII) [141]. A number of receptors have been proposed to bind SARS-CoV-2 [107]

There is a number of detailed recent reviews summarizing the role of platelets in immunological response [6, 15, 16, 18, 107, 134–139]; here, we provide only a couple of examples to show that platelets have prominent and not previously appreciated capabilities to regulate host defense and to regulate the immune functions of a variety of immune cells by a plethora of mechanisms and, thus, can be regarded as central players in host defense response, not only pertaining to bacterial but also to viral infections (Fig. 2). This non-hemostatic function of platelets likely plays an important role in responses to SARS-CoV-2, but the mechanisms involved are less understood as compared to those operating in host defense response to bacterial infections.

### Platelets and resolution of inflammation

**Fig. 3:**
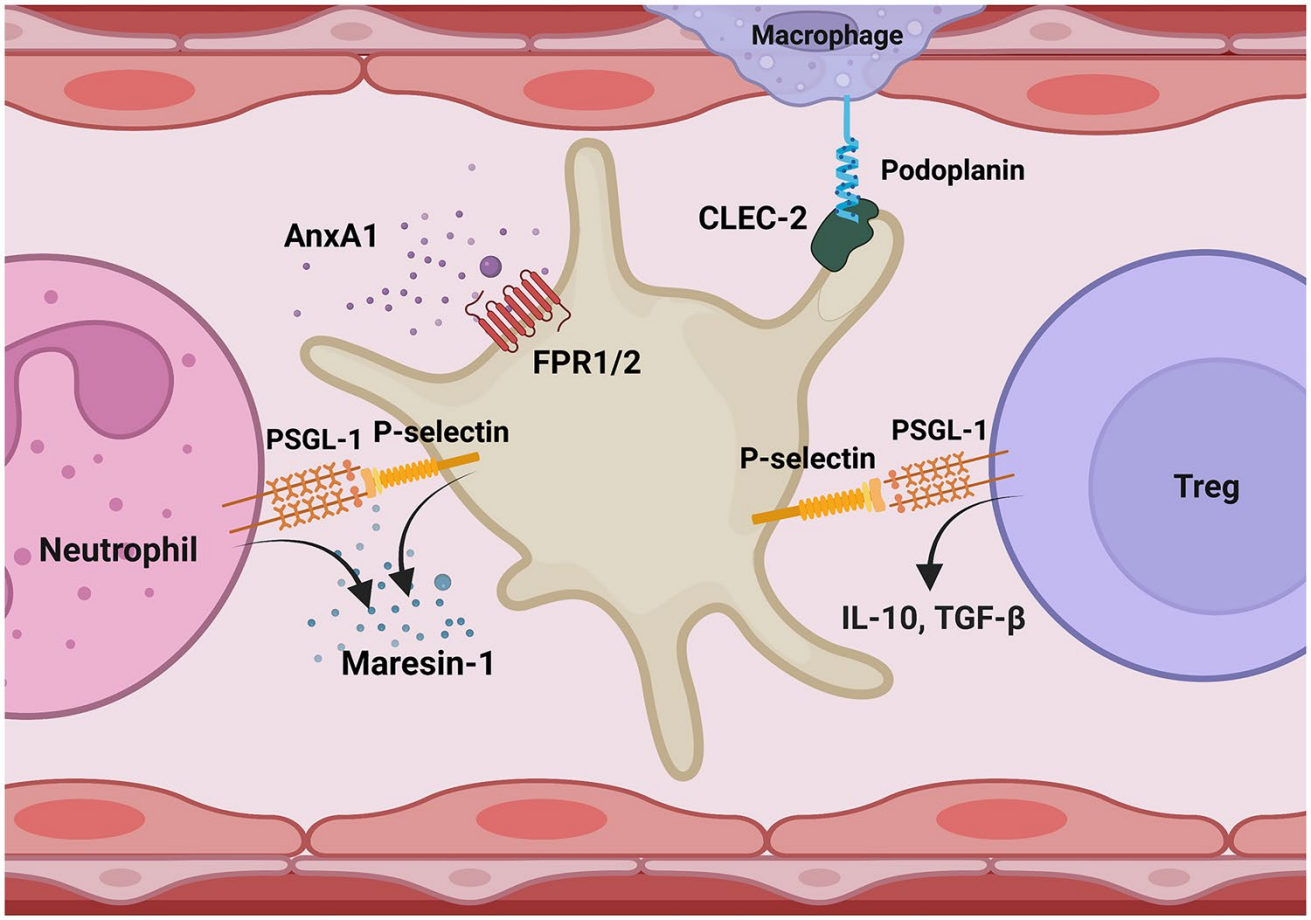
Non-hemostatic function of platelets; platelets in the resolution of inflammation. Activated platelets promote inflammation by a variety of well-known mechanisms, including the release of multiple inflammatory mediators which are synthetized (TXB_2_, and IL-1β) or released from platelet α and dense granules (e.g., TGFβ, RANTES, sCD40L) or by interacting with leukocytes via P-selectin or other receptors, which facilitates leukocyte transmigration, ROS production and phagocytosis [132]. On the other hand, platelets have recently emerged as regulators of inflammation resolution [134, 135, 152]. Platelets suppress pro-inflammatory macrophage phenotype via the interaction of CLEC-2 on platelets with podoplanin on macrophages [146], or the interaction of platelet P-selectin with PSGL-1 on *T*_reg_ cells that instruct *T*_reg_ cells to release the anti-inflammatory IL-10 and TGFβ [136]. Platelets also release pro-resolving mediators including Maresin-1 upon interactions with neutrophils [148]. Interaction of Annexin 1 (AnxA1) with its receptor on platelets increases platelet phosphatidylserine exposure, which promotes platelet phagocytosis by neutrophils, thereby driving active resolution [149]

The effective resolution of inflammation is necessary to maintain functional tissue homeostasis, since it prevents the progression of acute inflammatory response into persistent chronic inflammation, which may result in the onset or acceleration of the progression of multiple chronic diseases [142, 143]. For this reason, the initiation of an inflammatory response must be finely tuned with activation of its resolution [143]. Once the inciting agent has been effectively eliminated by the inflammatory response, the synthesis of pro-inflammatory mediators must be stopped, which warrants clearance of immune cells from the tissue via their apoptosis and phagocytosis by macrophages, as well as their entry into the lymphatic or systemic circulation [142]. Platelets actively participate in the resolution of the inflammatory response and a number of interesting mechanisms have been recently reported (Fig. 3). For example, sCD40L-activated platelets are recruited into the lungs as hetero-aggregates with *T*_reg_ lymphocytes formed by the direct interaction of platelet P-selectin with its receptor PSGL-1 on *T*_reg_ lymphocytes [136]. This physical interaction between *T*_reg_ cells and activated platelets is necessary to modulate the transcriptome and instruct *T*_reg_ lymphocytes to release the anti-inflammatory mediators IL-10 and TGFβ [136]. It is worth noting that the interaction of activated platelets with *T*_reg_ cells was also important for macrophage transcriptional reprogramming and macrophage polarization toward an anti-inflammatory phenotype, which effectively resolves pulmonary inflammation [136]. Furthermore, the *T*_reg_ lymphocytes recruited into the lungs by platelets could also promote macrophage efferocytosis during the resolution of inflammation, facilitating apoptotic cell clearance [144]. Interestingly, the use of P2Y_12_ receptor agonist clopidogrel-modified interaction of *T*_reg_ cells with platelets and restrained the proliferation of *T*_reg_ cells in a mouse model of sepsis [145].

It was also shown that platelet receptor CLEC-2 interaction with podoplanin expressed on inflammatory alveolar macrophages in the lungs in the mouse model of ARDS limited the severity of lung inflammation [146]. Moreover, platelets activated by collagen, via the release of prostaglandin E_2_ (PGE_2_), induced anti-inflammatory IL-10 release by monocytes or macrophages, which simultaneously restrained secretion of tumor necrosis factor α (TNFα) [147]. Finally, platelets were shown to release pro-resolving mediators that inhibited neutrophil infiltration into the tissue and exhibited organ-protective action [134]. In fact, the interaction of platelets with neutrophils enabled the generation of a mature form of pro-resolving mediator maresin 1 (MaR1) by platelet 12-lipoxygenase, which converted docosahexaenoic acid to 13S,14S-epoxy-maresin which was further processed to mature MaR1 by neutrophils [148]. In addition, platelets express receptors for some of the pro-resolving mediators, such as resolvin E1 receptor (ChemR32), resolvin D1 receptor (GPR32), or lipoxin A4 receptor (ALX), and platelet stimulation, with MaR1 led to the inhibition of pro-inflammatory mediator release from platelets [134]. Another interesting mechanism reported refers to annexin 1 (AnxA1), which increased platelet phosphatidylserine exposure promoting platelet phagocytosis by neutrophils, and, thereby, driving active resolution [149]. Last but not least, thrombin signaling via PAR-4 has also been argued to be of great importance in the regulation of inflammation resolution, since PAR-4deficiency impaired cardiac healing after myocardial infarction (MI) and increased cardiac hemorrhage potentiating post-MI cardiac dysfunction [150]. Interestingly, direct thrombin inhibitor dabigatran etexilate applied prior to CVB3 viral infection resulted in an increased number of viral genomes in the heart and more pronounced cardiac injury [151].

### Platelets and endothelial barrier integrity of the lungs

Platelets support the barrier function of pulmonary endothelium in healthy individuals [24, 153] while thrombocytopenia leads to the increased permeability of pulmonary vessels [24, 154–156]. In inflammatory conditions, although platelet activation was reported to contribute to lung injury and endothelial barrier disruption in acute lung injury [ALI], in acute respiratory distress syndrome (ARDS) [157] and in influenza virus infections [158], platelets can also maintain the endothelial barrier of the lungs via the mechanisms of inflammation-associated hemostasis [20–23, 159].

**Fig. 4:**
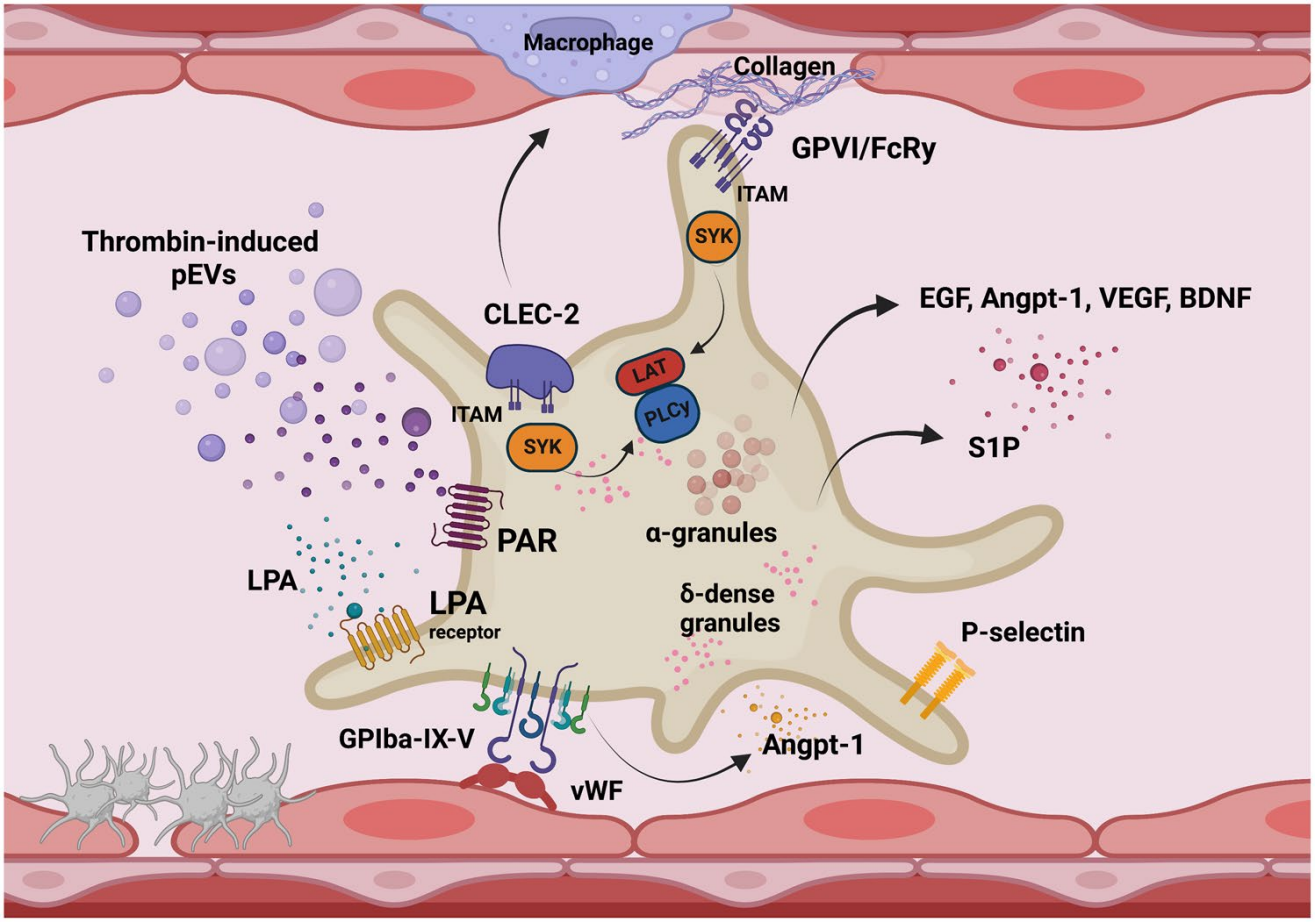
Non-hemostatic function of platelets; platelets maintain endothelial barrier integrity. Platelets maintain the molecular integrity of the adherens junctions by the constitutive release of growth factors. They include brain-derived neurotrophic factor (BDNF), epidermal growth factor (EGF), platelet-activating factor (PAF), sphingosine-1-phosphate (S1P), angiopoietin 1 (Angpt-1), and VEGF A (VEGF-A) [153]. The endothelial barrier can be also supported by lysophosphatidic acid (LPA) [162, 164] or by von Willebrand factor (vWF)-dependent release of Angpt-1 acting on endothelial receptor Tie-2 [169]. Platelet receptor CLEC-2 interacting with its ligand podoplanin on activated macrophages results in anti-inflammatory effects [146]. Activation of platelet CLEC-2 [172] and GPVI [173] receptors initiates intra-platelet ITAM-dependent signaling that results in protective effects on the endothelium [173]. Finally, thrombin-induced pEVs protect endothelial barrier integrity [174]

Unstimulated, quiescent platelets support endothelium barrier function in the lungs in the absence of injury or inflammation. Direct evidence for this was provided a few decades ago by an in-vivo study involving thrombocytopenic sheep that displayed increased pulmonary vascular permeability to protein compared to control animals [154]. In the same study, it was shown that isolated human platelets reduced radiolabelled albumin transfer across cultured bovine pulmonary artery endothelial monolayers in a way dependent on platelet number. Platelets also protected against edema formation in blood-perfused sheep lungs [155] and platelet depletion caused vessel abnormalities and hemorrhaging in the lungs of new-born mice [156]. The mechanism of constant endothelium support rendered by quiescent platelets depends upon the constitutive release of endothelial trophogens, including brain-derived neurotrophic factor (BDNF), epidermal growth factor (EGF), platelet-activating factor (PAF), sphingosine-1-phosphate (S1P), angiopoietin (Angtp-1), and vascular endothelial growth factor A (VEGF-A) [153] (Fig. 4). These trophogens signal through their respective receptors to maintain the stability of the intercellular junctions between neighboring endothelial cells [153]. The other possible mechanism could involve the release of lysophosphatidic acid (LPA) [160–163] acting via LPA receptor 4 (LPAR4), which assures the localization of VE-cadherin at endothelial cell boundaries [164]. These and other not yet uncovered platelet-dependent mechanisms maintain pulmonary endothelium integrity in the lungs of healthy individuals [20].

Platelets also protect the integrity of the endothelial barrier alongside an inflammatory response [165]. Therefore, thrombocytopenia may sensitize the pulmonary vasculature to more increased vascular leakage in the presence of an inflammatory or infectious stimulus [165]. Indeed, anticoagulation prior to infection with dabigatran or warfarin increased alveolar hemorrhage in mice infected with influenza A virus [166] and anticoagulation with dabigatran increased the numbers of coxsackievirus B3 virions in the infected tissue [151]. The endothelial barrier protective function of platelets is especially important in the lungs [167]. Freshly transfused platelets attenuated lung injury in the murine model of extracorporeal circulation (ECC)-induced systemic inflammatory response [23] and this protective effect of freshly transfused platelets was impaired in the presence of an antiplatelet drug, tirofiban. A similar effect was also observed in vitro, whereby the protective effect of thrombin-induced platelet releasate on the human microvascular lung endothelial barrier was significantly attenuated in the presence of the thrombin inhibitor dabigatran [168]. Moreover, via docking to the von Willebrand factor, platelets could prevent the leaks associated with leukocyte transmigration by releasing Angpt-1, which activates endothelial receptor Tie-2 signaling [169]. In fact, the docking mechanism could allow for significantly higher local concentrations of Angpt-1 (higher then Angpt-1 basal plasma level), and, thus, local activation of the protective Tie-2 signaling at distinct sites of platelet activation.

Another mechanism of pulmonary endothelium protection by platelets was uncovered in the murine model of ARDS and involves platelet receptor CLEC-2 which, by activation of its ligand podoplanin (expressed by inflammatory alveolar macrophages), inhibited the pro-inflammatory phenotype of these macrophages and protected against lung injury [146] (Fig. 4). Interaction of platelet CLEC-2 with macrophage podoplanin also limited the severity of sepsis [170], hampering immune cell infiltration and the inflammatory reaction at the site of infection [171].

## Conclusion

Platelets have been well recognized for their key role in classical hemostasis and antiplatelet therapeutic strategies represent a cornerstone therapy of cardiovascular diseases to prevent thrombosis. The lack of clinical benefit of antiplatelet therapy in COVID-19, despite clear-cut evidence for hyper-activated platelets and increased thrombosis risk, seems to suggest that the non-hemostatic role of platelets in host defense response may protect the host. In fact, platelets emerge as circulating blood elements which are vital in fighting the pathogen, sensing the pathogen, orchestrating innate and adaptive immune responses, regulating inflammation resolution as well as maintaining vascular integrity during multicellular host defense response that occurs intravascularly. Initially compromised pulmonary endothelial function could obviously precipitate a severe outcome of host defense response in the pulmonary circulation [175]. There is also limited knowledge that would allow to link the endothelial status of an individual (e.g. age-dependent endothelial dysfunction) with the ability of platelets to defend the host and to support endothelial barrier via the mechanisms of inflammation-associated hemostasis. Similarly, little is known about various platelet subpopulations that could theoretically afford distinct functions [176]. Nevertheless, the non-hemostatic functions of platelets seem to be of particular importance during the host response occurring in the pulmonary circulation, and the lung is the major organ that fails in severe and fatal COVID-19. Thrombocytopenia is associated with poor prognosis and the lung is a major source of megakaryocytes [177], as if to provide a reserve of new generations of platelets to secure the host defense response in the pulmonary circulation. Knowledge on platelet biology, is still far from complete, and we need to better understand platelet-dependent mechanisms involved in the host response, in the preservation of the endothelial barrier and in the vascular surveillance. This knowledge is necessary to better understand a possibly distinct intra-platelet mechanisms responsible for vaso-protection in comparison to mechanisms of classical hemostasis [139]. Therefore, further studies are needed to define to what extent current antiplatelet therapies affect non-hemostatic platelet functions and to design antiplatelet therapeutic strategies that inhibit the pro-thrombotic activity of platelets without affecting the non-hemostatic and vaso-protective function of platelets during multicellular host defense.

## Data Availability

The manuscript has no associated data.

## Abbreviations

SARS-CoV-2: Severe acute respiratory syndrome coronavirus 2
ARDS: Acute respiratory distress syndrome
ACE-2: Angiotensin-converting enzyme 2
NET: Neutrophil extracellular traps
RCTs: Recent randomized clinical trials
COX-1: Cyclo-oxygenase-1
GP: Glycoprotein
vWF: Willebrand factor
PAR: Protease-activated receptor
pEVs: Platelet-derived extracellular vesicles
CLEC-2: C-type lectin domain family 2
CLEC-5: C-type lectin domain family 5
CR: Complement receptor
CXCR: Cys-X-Cys motif chemokine receptor type
FcγRII: Fc gamma receptor IITLR: Toll-like receptor
NRLP3: Nucleotide-binding domain-like receptor protein 3
HMGB1: High-mobility group box 1 protein
TSP-1: Thrombospondin-1
EGF: Epidermal growth factor
PAF: Platelet-activating factor
VEGF A: Vascular endothelial growth factor A
BDNF: Brain-derived neurotrophic factor
S1P: Sphingosine-1-phosphate
TNFMI: Myocardial infarction
VTE: Venous thromboembolism
PE: Pulmonary embolism
PS: Phosphatidylserine
PF4: Platelet factor
TNF-α: Tumor necrosis factor α
IL: Interleukin
MCP-1: Monocyte chemoattractant protein 1
PRR: Pattern recognition receptors
ECC: Extracorporeal circulation
DAMPs: Damage-associated molecular patterns
VCAM-1: Vascular cell adhesion molecule 1
IgG: Immunoglobulin G
*T*_reg_: Regulatory lymphocytes T
ALI: Acute lung injury
Angtp-1: Angiopoietin
LPA: Lysophosphatidic acid
LPAR4: LPA receptor 4
ECC: Extracorporeal circulation
PDGF: Platelet-derived growth factor
bFGF: Basic fibroblast growth factor
TGFβ: Transforming growth factor β
eNOS: Endothelial nitric oxide synthase
